# Bioprintable Lung Extracellular Matrix Hydrogel Scaffolds for 3D Culture of Mesenchymal Stromal Cells

**DOI:** 10.3390/polym13142350

**Published:** 2021-07-18

**Authors:** Bryan Falcones, Héctor Sanz-Fraile, Esther Marhuenda, Irene Mendizábal, Ignacio Cabrera-Aguilera, Nanthilde Malandain, Juan J. Uriarte, Isaac Almendros, Daniel Navajas, Daniel J. Weiss, Ramon Farré, Jorge Otero

**Affiliations:** 1 Unitat de Biofísica i Bioenginyeria, Facultat de Medicina i Ciències de la Salut, Universitat de Barcelona, Casanova 143, 08036 Barcelona, Spain; bfalco86@gmail.com (B.F.); hector.sanz.fraile@hotmail.com (H.S.-F.); marhuenda.esther@gmail.com (E.M.); irenei.mendizabal@gmail.com (I.M.); ignaciocabrera.a@gmail.com (I.C.-A.); nmalandain@icmab.es (N.M.); isaac.almendros@ub.edu (I.A.); dnavajas@ub.edu (D.N.); rfarre@ub.edu (R.F.); 2 Centro de Investigación Biomédica en Red, Enfermedades Repiratorias, Monforte de Lemos 3-5, 28029 Madrid, Spain; 3 Department of Medicine, College of Medicine, University of Vermont, Burlington, VT 05405, USA; Juan.Uriarte@uvm.edu (J.J.U.); daniel.weiss@med.uvm.edu (D.J.W.); 4 Institut d’Investigacions Biomèdiques Agustí Pi i Sunyer, Roselló 149, 08036 Barcelona, Spain; 5 Institut de Bioenginyeria de Catalunya, Baldiri Reixac 10-12, 08028 Barcelona, Spain

**Keywords:** 3D Bioprinting, hydrogels, extracellular matrix, mesenchymal stromal cells, tissue engineering, acute lung injury

## Abstract

Mesenchymal stromal cell (MSC)-based cell therapy in acute respiratory diseases is based on MSC secretion of paracrine factors. Several strategies have proposed to improve this are being explored including pre-conditioning the MSCs prior to administration. We here propose a strategy for improving the therapeutic efficacy of MSCs based on cell preconditioning by growing them in native extracellular matrix (ECM) derived from the lung. To this end, a bioink with tunable stiffness based on decellularized porcine lung ECM hydrogels was developed and characterized. The bioink was suitable for 3D culturing of lung-resident MSCs without the need for additional chemical or physical crosslinking. MSCs showed good viability, and contraction assays showed the existence of cell–matrix interactions in the bioprinted scaffolds. Adhesion capacity and length of the focal adhesions formed were increased for the cells cultured within the lung hydrogel scaffolds. Also, there was more than a 20-fold increase of the expression of the CXCR4 receptor in the 3D-cultured cells compared to the cells cultured in plastic. Secretion of cytokines when cultured in an in vitro model of lung injury showed a decreased secretion of pro-inflammatory mediators for the cells cultured in the 3D scaffolds. Moreover, the morphology of the harvested cells was markedly different with respect to conventionally (2D) cultured MSCs. In conclusion, the developed bioink can be used to bioprint structures aimed to improve preconditioning MSCs for therapeutic purposes.

## 1. Introduction

Mesenchymal stromal cells (MSCs) are multipotent stem cells with self-renewal capability which can be derived from various tissues [1], including the lung [2,3]. It has been shown that MSCs mediate immunosuppression in animal models and in humans by secreting paracrine factors such as interleukin (IL)-10, prostaglandin (PG)-E2, and IL receptor antagonist (IL1RA) [4]. Also, they can regulate inflammatory processes and repair damaged cells and tissues by adhering to inflammatory sites and secreting extracellular matrix (ECM) proteins and other bioactive molecules to stimulate tissue regeneration [5]. MSCs have been proposed as cell therapy for a wide range of diseases [6], with their therapeutic potential extensively reported in applications such as treatment of autoimmune diseases [7]. However, its usefulness is still controversial in some other areas such as cancer [8], where MSCs present both pro- and antitumorigenic effects. Intensive research efforts are currently focused on identifying the secreted factors having a positive outcome in order to use them instead of cell therapy [9]. In the case of lung diseases, although the main hypothesis is that most benefits induced by the use of MSCs for treatment come from their paracrine effect, the determination of the technical issues related to cell culture previous to administration is still an open issue [10]. Accordingly, a considerable effort is now currently devoted to precondition the MSCs by optimizing culture conditions to improve their therapeutic potential before infusion [11,12]. Most strategies for pre-conditioning MSCs involve the use of routine tissue culture in which different cytokines, growth factors, hormones [13], or pharmacological agents [14] are supplemented to the cells. Moreover, the use of bioreactor systems to provide a temporally and spatially controlled environment to the cells is a growing strategy in the culture of MSCs [15]. However, although it is known that MSCs sense and respond to the biophysical stimuli [16], most conventional bioreactors are not specifically designed to account for the mechanosensitive capacities of MSCs and their potential to interact with the ECM network and the neighboring cells [17]. Physical properties of the microenvironment, such as stiffness, have been shown to regulate several physiological responses in different cell types, including MSCs secretion of a wide range of cytokines [18]. Recently, it has been reported that culturing lung resident MSCs on ECM while simultaneously subjecting them to cyclic stretch mimicking breathing improved their in vivo therapeutic effect in a rat model of ventilator-induced lung injury [19]. 

In the case of the lung, it has been hypothesized that the key for successful implementation of MSC-based cell therapies could lie in an improvement of the physical microenvironment of the scaffolds where cells are cultured [18]. It has been shown that the culture of MSCs by using specifically-designed scaffolds impacts on their adhesion pattern, regulates cell polarity, affects migration, enhances endogenous ECM expression, and promotes the secretion of anti-inflammatory mediators [20,21]. Physical factors like topography and stiffness of the scaffolds [22], as well as oxygen tension [23], have been found to regulate MSC morphology, proliferation, multi-lineage differentiation potential, anti-inflammatory response, and cell activities for repairing dysfunctional organs. In the specific case of the secretion of immunomodulatory mediators, it has been shown that culturing MSCs in 3D scaffolds impacts on their immunophenotype [24], and that fibrous characteristics of the scaffolds (fiber size, length, and alignment) modulate the paracrine function of MSCs from different sources cultured in the scaffolds [25]. Interestingly, decellularized tissues [26] and fibroblast-derived matrices [27] are promising scaffolds for culturing lung MSCs in a 2D microenvironment more closely mimicking the conditions found in the lung. However, the native niche of tissue resident MSCs is three-dimensional, thus culturing cells in 3D scaffolds has shown a major impact in cell behavior [28,29]. With the development of 3D bioprinting technologies, cell-laden hydrogels can be nowadays used as bioinks to 3D-print layer-by-layer the scaffolds [30]. Bioprinting offers a high-throughput method for fabrication of 3D in vitro cultures, offering higher accuracy, resolution and precision over conventional 3D culture methods in pharmaceutical manufacturing [31] and drug discovery [32]. ECM from animal tissues can be obtained from decellularization of the native tissues with detergents [33], and then pulverized and reconstituted in the form of a hydrogel suitable for 3D cell culture [34]. Following this general approach, hydrogels from decellularized porcine lungs had been developed [35] and investigated for the culture of human bone-marrow MSCs. Moreover, hydrogels from human lungs from patients with different diseases [36] have been developed and their viscoelasticity shown to depend on the pathology of the donor.

Stiffness of the scaffold in 3D culture has been shown to influence the direction of differentiation of MSCs [37], but the impact on their immunomodulatory properties is still to be elucidated. Stiffness of ECM-derived hydrogels is lower than the stiffness of the native tissue, but it can be modified by chemical processes, such as genipin or photo-crosslinking [38,39]. However, some of these interventions could be potentially toxic for cells or alter their response, as well as to modify the structure and composition of the scaffold. An alternative is to change the concentration of the ECM hydrogel, as it is directly related with the stiffness of the resulting scaffold. In the present work, the biofabrication of decellularized porcine lung ECM hydrogels (L-ECM), with no addition of other crosslinkers, was optimized to bioprint 3D scaffolds for culturing and preconditioning lung-resident MSCs (L-MSCs). Stiffness was tuned by changing the concentration of the ECM powder in the hydrogel preparation. The physical characteristics of L-ECM hydrogels (elastic and viscoelastic properties) were measured and their effects on L-MSCs in 3D culture were assessed by analyzing the changes in their adhesion capacity, expression of CXCR4 receptor and immunomodulatory cytokines secretion when tested in an in vitro model of acute respiratory distress syndrome. Therefore, the specific aim of this work was twofold. First, to develop and characterize an optimized bioprintable hydrogel made from lung ECM tuned to have physical properties closer to those found in the native lung ECM. Second, to test the hypothesis that 3D culturing L-MSCs into the bioprinted L-ECM hydrogels is a good strategy to better understand different factors affecting their therapeutic potential.

## 2. Materials and Methods

All the reagents were purchased from Sigma-Aldrich, St. Louis, MO, USA or ThermoFisher Scientific, Waltham, MA, USA, unless otherwise specified.

### 2.1. Preparation of Lung Extracellular Matrix (L-ECM) Hydrogels for Bioprinting

Optimized L-ECM hydrogels for L-MSC culturing were obtained by decellularizing porcine lungs, preparing a pregel bioink from the decellularized lung ECM, and bioprinting 3D constructs with customized shape and size by means of a bioprinter using support sacrificial material.

#### 2.1.1. Porcine Lung Decellularization

Porcine lungs from three different animals were obtained from a local slaughterhouse and decellularized by following the protocol from [35] with slight adaptations: in brief, lungs were perfused through the trachea and the vasculature with 0.1% Triton X-100, sodium deoxycholate, DNase and 1 M sodium chloride, with intermediate perfusion with distilled water and PBS for rinsing purposes. To verify the absence of cell DNA after the process of decellularization, 4′,6-diamidino-2-phenylindole (DAPI) fluorescence staining was used. Samples of every decellularized lung were immersed in optical cutting temperature compound (OCT, Sakura), frozen at −80 °C, and subsequently cryosectioned into 15 µm slices (Cryomicrotome HM 560, ThermoFisher Scientific, Waltham, MA, USA). The cryosections were rinsed with PBS to remove the OCT and then maintained for 10 min with 1 mg/mL DAPI solution for staining. To assess the effectiveness of decellularization, total genomic DNA was isolated using the PureLink Genomic DNA kit (ThermoScientific, Waltham, MA, USA) from native and decellularized scaffolds (n = 4 for each decellularized lung) following the manufacturer’s instructions. The total amount of DNA was quantified using spectrophotometry and normalized to the sample tissue dry weight.

#### 2.1.2. Preparation of L-ECM and Collagen Bioinks

Decellularized lungs were sliced and frozen at −80 °C, freeze-dried (Telstar Lyoquest-55 Plus, Terrassa, Spain), and pulverized into micron-sized particles at −180 °C by using a cryogenic mill (6755, SPEX, Metuchen NJA) for 5 min at maximum speed. The resulting powder was digested at a concentration of 20 mg/mL in a 0.01 M HCl solution (pH = 2) with pepsin from porcine gastric mucosa (1:10 concentration) under magnetic stirring at room temperature for 16 h. The resulting (pregel) solution was then pH-adjusted to 7.4 (±0.4) by using 0.1 M NaOH and PBS 10X and frozen at −80 °C for subsequent use. For the preparation of the bioink, the pregel was thawed to 4 °C, centrifuged at 1000× *g* for 5 min to remove air bubbles, and diluted to the desired concentration with PBS 1X. Pregels not diluted (20 mg/mL) will be referred to as high-concentration L-ECM (HC-L-ECM) while pregels diluted 1:1 (10 mg/mL) will be referred as low-concentration L-ECM (LC-L-ECM).

For ultrastructure and mechanical properties comparison, telocollagen hydrogel was used. Rat-tail type I collagen was extracted by following the protocol in [40] and then solubilized in 0.02 N acetic acid (pH = 3.2) at 4 °C. Pregel was prepared by adjusting the pH to 7.4 (±0.4) with 1 M NaOH for a final protein concentration of 7.5 mg/mL and will be referred to as COL1. For gelification, the pregel was incubated at 37 °C for 30 min.

#### 2.1.3. Bioprinting 3D Hydrogels

**Figure 1:**
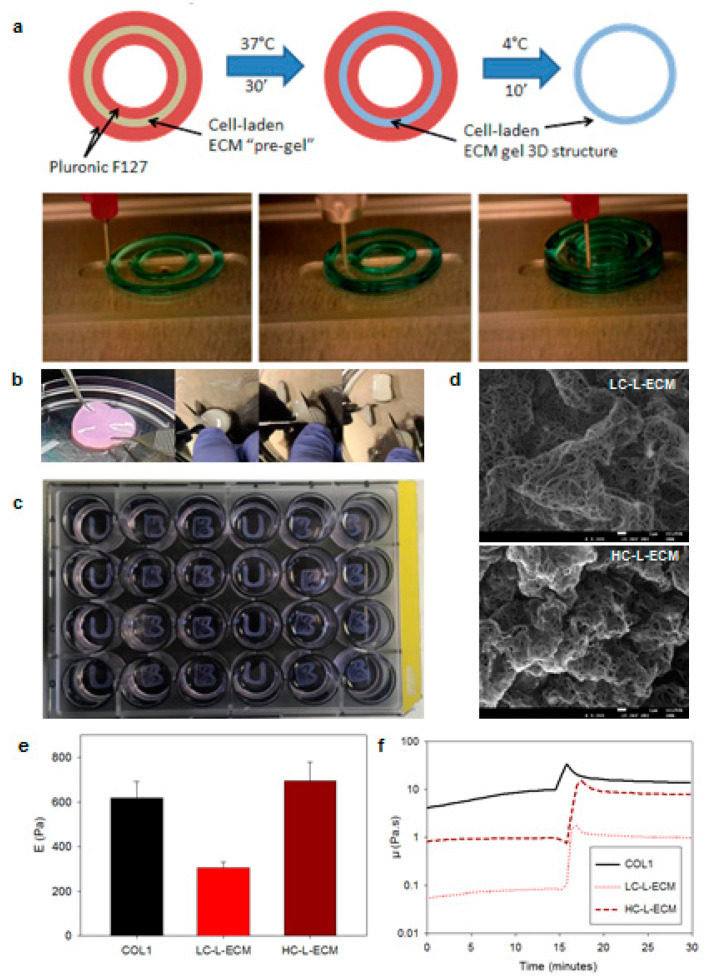
Fabrication and characterization of the lung extracellular matrix (L-ECM) scaffolds. (**a**) Scheme and photographs of the bioprinting process using F-127 as a sacrificial layer. L-ECM was printed in liquid phase. After gelification of the cell-laden hydrogel, the pluronic structure was dissolved. (**b**) Macroscopic images of the high concentration (HC)-L-ECM structures showing structural integrity allowing manipulation with tweezers and to be cut with a scalpel. (**c**) Scaffolds 3D-bioprinted in multiple shapes in a standard p24 well-plate. (**d**) Representative scanning electron microscope (SEM) images of the low (left) and high (right) concentration lung hydrogels ultrastructure. Scale bar = 1 µm (**e**) Quantification of the apparent Young’s modulus of the different hydrogels with the atomic force microscope. (**f**) Viscosity (*µ*) of the different hydrogels when temperature was varied from 4 °C to 37 °C, at time 15 min, measured by rheometry.

A droplet-printing cartridge of the 3D bioprinter (3Ddiscovery, RegenHU, Villaz-St-Pierre, Switzerland) was filled with the L-ECM pregel solution and maintained at 4 °C during all the printing process. A secondary printing cartridge was filled with Pluronic F127 gel (40% *v*/*v* in PBS) at room temperature. L-ECM was printed at approximately 2 bar pressure using a nozzle of 300 µm (RegenHU, Villaz-St-Pierre, Switzerland) and a microvalve aperture time of 1 ms, while F127 was printed at approximately 4.5 Bar using a needle of 250 µm (Nordson EFD, Westlake, OH, USA). The 3D structures were then constructed layer-by-layer by alternatively printing an F127 layer, which served as a template, and an L-ECM pregel layer which filled the F127 template layer. After the last layer was printed, the 3D structures were incubated at 37 °C for 30 min to form the hydrogel. The F127 component was subsequently dissolved by immersing the structure in culture media at 4 °C for 10 min (Figure 1a). Telocollagen (COL1) acellular structures were bioprinted by using the same protocol.

### 2.2. Characterization of L-ECM Hydrogels

Three-dimensional L-ECM hydrogels were structurally characterized by scanning electron microscopy (SEM) and their mechanical properties were measured at macro- and micro-scales by rheometry and atomic force microscopy (AFM), respectively.

#### 2.2.1. Scanning Electron Microscopy (SEM) Imaging of L-ECM Hydrogels

Microstructure characterization with scanning electron microscopy (SEM) was carried out by adapting the protocols in [35,41]. Briefly, COL1, HC-L-ECM and LC-L-ECM thin structures were fixed for 48 h in 4% PFA in PBS and then washed three times in 0.1 M phosphate buffer (PB) for 10 min. Resulting samples were incubated in 4% osmium tetroxide for 90 min followed by successive washes in deionized water until there was absence of osmium tetroxide. Then, samples were dehydrated in increasing concentrations of ethanol solutions and preserved in absolute ethanol at 4 °C and critical point dried using an autosamdri-815 critical point dryer (Tousimis, Rockville, MD, USA). Samples were mounted using conductive adhesive tabs (TED PELLA, Redding, CA, USA) for imaging and were carbon coated before imaging with a JSM-6510 (JEOL, Tokyo, Japan) scanning electron microscope at 15 kV. ImageJ software (National Institutes of Health, Bethesda, MD, USA) was used to process the SEM images. Density of fibers per area was obtained by applying a threshold to isolate the fibers in a specific plane to quantify them, while diameter of fibers was estimated as the average of ten randomly-selected fibers in three different zones of each sample.

#### 2.2.2. Rheological Characterization of L-ECM Hydrogels

COL1, LC-L-ECM, and HC-L-ECM hydrogel rheological properties were measured by using a HAAKE RheoStress 1 rheometer (ThermoFisher, Waltham, MA, USA) with 35 mm serrated parallel plate geometry. Hydrogels were neutralized just before each mechanical testing was performed. Then, a pregel solution was loaded into a Peltier plate set at 4 °C and with a distance between plates of 200 µm. Storage (*G′*) and loss (*G″*) moduli, and viscosity (*µ*, defined as the *G′/G″* at each angular velocity) were measured at constant 0.1 Hz with a strain of 5%. The temperature of the plates was kept constant at 4 °C for 15 min, then increased to 37 °C and subsequently held constant for 15 min.

#### 2.2.3. Micromechanical Properties Measurement of L-ECM Hydrogels

Micromechanical properties of COL1, LC-L-ECM and HC-L-ECM acellular hydrogels were measured by atomic force microscopy (AFM). Specific geometries for the measurements were bioprinted as 10 mm × 10 mm × 0.1 mm layers attached on top of positively charged glass slides. All the measurements were performed inside a bath with PBS at 37 °C. Three samples were prepared for each concentration of the hydrogel. Measurements were conducted with a custom-built AFM mounted on an inverted optical microscope (TE2000; Nikon, Tokyo, Japan) equipped with V-shaped silicon nitride cantilevers (0.01 N/m nominal spring constant) ended with a 6 μm radius spherical borosilicate bead (Novascan Technologies, Ames, IA, USA). The apparent Young’s elastic modulus (*E*) was computed from the force-displacement curves by adjusting the Hertz model as described in [42]. The micromechanics of each sample were probed in five randomly selected zones. Five force curves (1 Hz and 15 µm amplitude) in four points randomly selected and separated ~50–100 µm from each other were recorded in each zone. Micromechanical stiffness of each sample was characterized as the average from the different curves recorded in the sample. The value for each hydrogel was calculated as the average of the three samples’ measurement.

### 2.3. Bioprinting and 3D Culturing Lung Mesenchymal Stem Cells into L-ECM Hydrogels

L-MSCs were isolated from rats, included into L-ECM pregel and 3D bioprinted and cultured within the 3D scaffolds for 7 days. Spatial distribution of L-MSC embedded into the 3D L-ECM constructs was assessed.

#### 2.3.1. Isolation of L-MSCs

L-MSCs were isolated from rat lungs adapting a protocol from [1] and approved by the Ethical Committee For Animal Research of the University of Barcelona. Briefly, Sprague-Dawley rats (250 g) were anesthetized intraperitoneally with urethane (10 mL/kg animal) and sacrificed by exsanguination through the abdominal aorta. PBS 1X (50 mL) was perfused into the right ventricle of the beating heart after cutting the left atrium. Lungs were excised en bloc with the heart and cut in small pieces before being washed in cold PBS 1X. Subsequently, 1 mL of 250 U/mL of collagenase I solution prepared in DMEM with 10 mM of HEPES was used to keep the pieces humidified while they were finely excised. Subsequently, the mixture was resuspended in 10 mL of collagenase I solution and digested for 1 h at 37 °C under constant motion. Gross remnants were discarded by 250 µm filtration and the supernatant was centrifuged at 400 g for 10 min to pellet the cells (Rotina 380R, Hettich, Tuttlingen, Germany). Red blood lysis buffer (RBC) (BioLegend, San Diego, CA, USA) was added and incubated with the cells at 4 °C for 7 min and the reaction was stopped with PBS 1X. Finally, cells were centrifuged at 350 g for 5 min and cultured on conventional plastic vessel T-75 (Techno Plastic Products AG, Trasadingen, Switzerland) with DMEM supplemented with 10% FBS antibiotic/antimycotic solution and incubated with 5% CO_2_ balanced-air incubator at 37 °C. After three days, medium was replaced to discard all non-adhered cells further and trypsinized for 7 min with TripLE express trypsin before they reach confluence. MSC phenotype for the isolated cells was assessed via characterization of cell surface markers (CD29, CD44H, CD45, CD11b and CD90) (BioLegend, San Diego, CA, USA) and analyzed by FACS CANTO II (BD Bioscience, San Jose, CA, USA). A differentiation assay kit (R&D Systems, Minneapolis, MN, USA) was used to differentiate cells to adipocyte, osteocyte and chondrocyte according to the manufacturer’s instructions. Briefly, L-MSCs were plated in a 24-wells plate until reached 100% for adipocyte and 70% confluence for osteocyte differentiation assays. Regarding chondrocyte differentiation, L-MSCs were pelleted in a 15 mL falcon tube. Afterwards, for each specific differentiation media was changed every 3–4 days over 3 weeks and the cells subsequently stained with specific linage markers supplied in the kit: FABP4 (adipocyte), hOsteocalcin (osteocyte) and hAgreccan (chondrocyte).

#### 2.3.2. Bioprinting and Culturing of Lung Mesenchymal Stem Cells into 3D L-ECM Hydrogels

Pregel was then mixed with the cells suspended in culture media at a ratio 10:1 *v*/*v* while maintaining the pregel solution at 4 °C (liquid phase). All experiments were conducted with cells at passages 3–7. Isolated and expanded L-MSCs were trypsinized and resuspended in culture medium (3 × 105 cells/mL), mixed with the L-ECM pregels as described in Section 2.1.3 (n = 5 for each concentration) and 3D bioprinted into cylindrical structures of 1.5 mm of thickness and 1 mm of radius for hydrogel contraction assay, and casted into standard petri dishes for harvesting purposes. To form the cell-laden hydrogel, the pregel was incubated at 37 °C for 30 min. Cells were cultured for 7 days into the 3D structures while changing the culture medium every 3 days. Control L-MSCs were maintained for culture in parallel in standard tissue culture plastic (TCP).

#### 2.3.3. Distribution and Viability of L-MSC Embedded into 3D L-ECM Hydrogel

For histological analysis of the cells within the hydrogels, very thin structures (~100 µm) were bioprinted on top of P6 MatTek (MatTek Corp., Ashland, MA, USA) wells. Samples were then stained for DNA (NucBlue) and F-actin (phalloidin) and imaged with a confocal microscope (TI-HUBC, Nikon, Tokyo, Japan) with a 60× objective.

Viability analysis within the hydrogels was done by live imaging of L-ECM hydrogel structures at day 7. A Live/Dead viability kit (Invitrogen) was used following the manufacturer’s instructions. Briefly, hydrogels were washed twice with PBS 1X and incubated with 2 µM calcein AM (live cells, green) and 4µM EthD-1 (dead cells, red) for 20 min in the incubator. Structures were then washed again twice in PBS 1X and immediately imaged with an inverted microscope (TI-HUBC, Nikkon Inst., Amsterdam, The Netherlands) attached to a motorized stage and equipped with a charge-coupled device camera, and confocal images of each well were obtained using a 10× objective. Z stacks obtained from the live/dead staining with the confocal microscope were used to assess the 3D spatial distribution of L-MSCs within the structures.

### 2.4. Effects of 3D Culturing into L-ECM Hydrogels on L-MSC Scaffold Contraction and Adhesion Properties

Cell contraction of the L-ECM hydrogels was assessed along 7 days of 3D culture. In addition, how such cell preconditioning modulated the adhesion and immunomodulatory capacity of cells harvested from the 3D culture was evaluated.

#### 2.4.1. Assessment of the Contraction of L-ECM Hydrogels in the 3D Cultures

L-ECM hydrogels contraction was quantified every 72 h using macroscopic image analysis. Free-floating L-ECM hydrogels were imaged with a digital microscope (Dino-Lite Edge, AnMo Electronics Corp., New Taipei, Taiwan). Briefly, the culture media was aspirated with a pipette to let the structure collapse towards the surface and take the image without error induced by floating structure tilting. The culture media was then immediately re-added to the culture plate. Quantification was carried out as described in [43] and results were expressed as fold-change respect to the contraction of acellular L-ECM hydrogels.

#### 2.4.2. Adhesive Properties of L-MSCs Harvested from the 3D Scaffolds

Cells were harvested from the hydrogel 3D cultures at day 7 by digesting the L-ECM structures with collagenase. The wells of each hydrogel were washed twice with PBS 1X and incubated for 10 min with Trypsin and the reaction was stopped with FBS-supplemented DMEM. Small pieces were cut and incubated with 1000 U/mL collagenase I solution, keeping constant agitation at 37 °C for 20 min. This step allowed digestion of the ECM scaffold and release of the cells. Finally, the process was stopped and cells were collected with full supplemented DMEM, filtered through pores of 100 µm and centrifuged at 400× *g* for 10 min. Cells were counted and resuspended for subsequent experiments.

The expression of C-X-C chemokine receptor type 4 (CXCR4) in harvested and control cells was quantified by qRT-PCR. RNA extraction was performed using the RNeasy Mini Kit (Qiagen, Hilden, Germany) following the manufacturer’s instructions and quantified with a microvolume spectrometer (Colibri, Titertek Berthold, Pforzheim, Germany). Reverse transcription from RNA samples was done using a TaqManTM Reverse Transcription kit (Invitrogen) and a thermal cycler (Techne TC-3000G). Changes in the expression of CXCR4 were measured by following the TaqManTM Fast Advanced Master Mix kit (Applied Biosystems) in the StepOne Real-Time PCR System (Applied Biosystems) by using the primer Rn 01483207 (ThermoScientific, Waltham, MA, USA). Relative CXCR4 gene expression was normalized to the expression of peptidylproplyl isomerase A (PPIA) gene (housekeeping gene) by applying the 2^−∆∆Ct^ method [44] to compute the fold changes in expression of these genes compared to baseline.

Assessments of cytoskeletal structures and of physical adhesion were conducted in the harvested and control cells by seeding them onto specific well-plates for optical imaging (Mattek, Ashland, MA, USA) and allowed to attach to the plate for 2 h. At this time point, well-plates were washed twice with ice-cold PBS 1X to remove non-adhered cells. Plates were immediately fixed with PFA 4% in PBS 1X for 15 min. After repeated washes, cells were permeabilized with 0.2% Triton X-100, blocked with a 10% FBS solution and incubated overnight with anti-paxillin rabbit monoclonal (Y113, ab32084, Abcam, Cambridge, UK). Cells were then incubated for 2 h with the secondary antibody Alexa Fluor 488 anti-rabbit (ab150077, Abcam, Cambridge, UK), counter-stained for nuclei (NucBlue, ThermoScientific, Waltham, MA, USA) and F-actin (phalloidin, Thermoscientific, Waltham, MA, USA) and imaged with the confocal microscope. Acquired images were randomly blinded and analyzed. For cell adhesion quantification, five images from each sample were obtained with a 20× objective by composite generation of four adjacent fields. Images were analyzed by counting the nuclei in ImageJ software using the tools watershed and analyze particles. For focal adhesions assessing, high magnification images (60× objective) of at least 20 single cells for each condition were acquired and quantified according to [45]. Briefly, the lengths of the focal adhesions were quantified for four representative adhesions in each cell at its edge.

#### 2.4.3. Immunomodulatory Properties of L-MSCs Harvested from the Scaffolds

Human primary small airway epithelial cells (HSAECs, ATCC, Manassas, VA, USA) were cultured and expanded in airway cell basal medium following manufacturer instructions. L-MSCs harvested from the 3D cultures in HC-L-ECM and LC-L-ECM, or control L-MSCs (cultured in 2D) were co-cultured in direct contact with HSAECs at a ratio L-MSCs:HSAECs of 1:4 in 12-wells plates with HSAECs medium. After 16 h of seeding, the cultures were washed twice with PBS 1X and replaced by serum-free media with either 20 µg/mL of lipopolysaccharides (LPS) or serum-free media for control, following a protocol for an in vitro model of acute respiratory distress syndrome (ARDS) adapted from [46]. After 40 h of co-culture, inflammatory (IL-6 and IL-8) cytokines were measured from the supernatants by enzyme-linked immunosorbent assay (ELISA) following the DuoSet ELISA Development kit (R&D Systems, Minneapolis, MN, USA) instructions.

#### 2.4.4. Morphology Analysis of Cells Harvested from the Hydrogels

Cells were harvested from the scaffolds as described for the adhesion experiments, reseeded onto specific well-plates for optical imaging (Mattek, Ashland, MA, USA) and allowed to attach to the plate for 4 h and then stained for nuclei and cytoskeleton as described previously. Optical images of 20 cells for each group were acquired at high magnification (60× objective). Eccentricity of the cells was computed by adjusting an ellipse to the polygon described by the cell using ImageJ software, as described in [47].

### 2.5. Statistical Analysis

Data are expressed as mean ± SE. One-way analysis of variance (ANOVA) tests were performed to compare the changes induced by the different concentrations of the hydrogels, and to compare the effects observed when cells were cultured in 3D and 2D. Statistical significance was considered at *p*-values < 0.05.

## 3. Results

### 3.1. Structural Characterization of 3D Bioprinted L-ECM Hydrogels

DAPI stained samples showed no visible nuclei in the decellularized tissues, and DNA quantification confirmed that decellularization was satisfactory: 17 ± 8 ng of DNA per mg of dry tissue, a value below the currently accepted threshold (50 ng/mg) [48]. Macroscopic photographs of the developed constructs of HC-L-ECM hydrogels (Figure 1b) showed that the structures were consistent enough to be manipulated with tweezers and to be cut with a scalpel. Also, they could be 3D-bioprinted (Figure 1c). Analysis of the SEM images (Figure 1d) showed the expected fibrillary architecture of the scaffold, without significant fiber size differences among the different ECM concentrations in the preparation of the hydrogel (126 ± 12 nm for COL1, 139 ± 15 nm for LC-L-ECM, and 131 ± 15 nm for HC-L-ECM), which are quite similar to the reported values for decellularized pig lung tissue fibrils [35].

### 3.2. Rheological Properties

Mean values for the viscosity of the lung ECM pregels was much lower than collagen pregels (Figure 1f): 0.07 Pa·s for the LC-L-ECM and 0.93 Pa·s for the HC-L-ECM while the viscosity of collagen pregel varied from 4.12 Pa·s at time 0 to 9.86 Pa·s after 15 min at 4 °C. It is interesting to note that viscosity at 4 °C remained constant for ECM pregels while the opposite occurred with collagen pregels. Gelation was very fast in all cases once the temperature reached 37 °C, around 2 min for the LC-L_ECM while gelation of the HC-L-ECM and telocollagen hydrogels occurred in about 1 min. Once gelated, values of *G′* and *G″* for the HC-L-ECM were in the same range as collagen hydrogels (*G′* = 4.91 Pa, *G″* = 1.85 Pa for the ECM hydrogels, *G′* = 8.54 Pa, *G″* = 3.54 Pa for collagen), while the rheological properties of LC-L-ECM were one order of magnitude lower (*G′* = 0.52 Pa, *G″* = 0.39 Pa).

### 3.3. Micromechanical Properties

Micromechanical properties of HC-L-ECM hydrogels were comparable to telocollagen hydrogel properties (*E* = 0.69 ± 0.08 kPa for the ECM and *E* = 0.62 ± 0.07 kPa for the collagen hydrogels), while the Young’s elastic modulus of LC-L-ECM hydrogels was comparable with medium-to-low concentration collagen I hydrogels (*E* = 0.31 ± 0.09 kPa) [49].

### 3.4. Cell Distribution and Viability in 3D Hydrogel Culture

**Figure 2:**
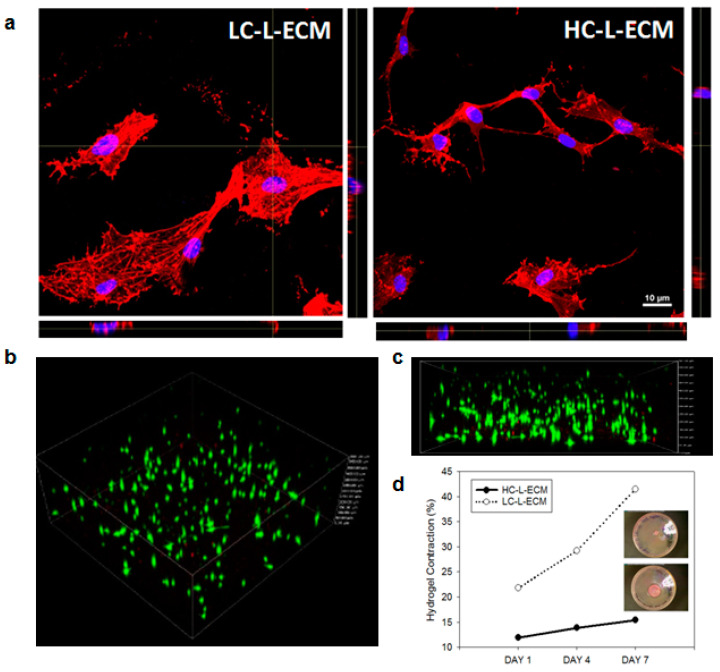
Lung-resident mesenchymal stromal cells (L-MSCs) in 3D lung ECM hydrogels. (**a**) 3D confocal images of the L-MSCs within the hydrogel scaffolds. (**b**) Three-dimensional live/dead image and (**c**) Z-distribution of the bioprinted cells within the 3D hydrogel structure. (**d**) Evolution of contraction of the cell-laden bioprinted low-concentration L-ECM (LC-L-ECM) and high-concentration L-ECM (HC-L-ECM) structures at days 1, 4 and 7.

L-MSCs bioprinted and 3D-cultured within both LC-L-ECM and HC-L-ECM hydrogels (Figure 2a) showed a spread morphology within the scaffolds. Cells exhibited high viability after 7 days of culture (Figure 2b), and *Z*-distribution (Figure 2c) showed that cells were reasonably distributed and not deposited at the bottom of the structure after the processes of bioprinting and gelification.

### 3.5. Cell Contraction within the 3D Hydrogel

Both types of structure were significantly contracted during the 3D cell culture, indicating that L-MSCs not only were alive but they actively interacted with the scaffold (Figure 2d). As expected, the measured contraction of the softer structures was more than two-fold higher than the contraction of the stiffer hydrogels, resulting in more than 40% of area reduction after 7 days in 3D culture.

### 3.6. Adhesion of Cells Preconditioned by 3D L-ECM Hydrogel Culturing

**Figure 3:**
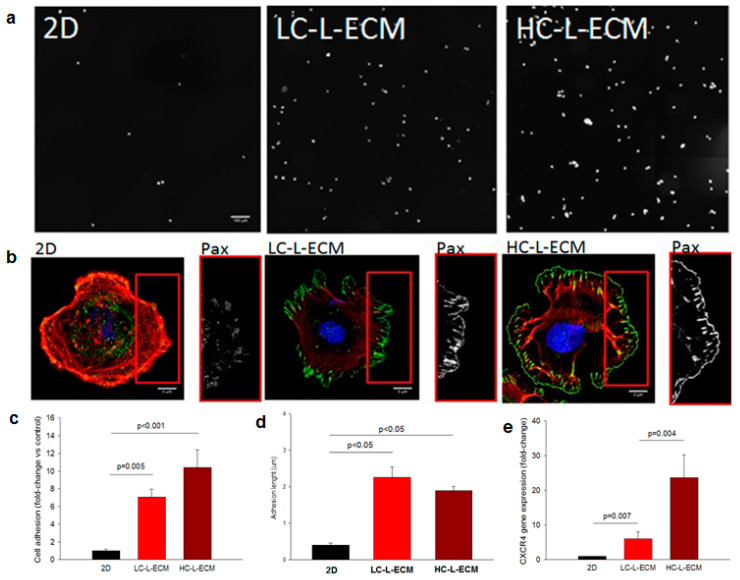
L-MSCs harvested from the 3D-cultured hydrogels. (**a**) Cell adhesion quantification: representative images of cells harvested from the LC-L-ECM and HC-L-ECM structures compared to the 2D-cultured control cells used (2 h after seeding). Scale bar = 100 µm (**b**) Representative images of the focal adhesion quantification from the actin (red) and paxillin (green) staining, colocalization is shown in white indicating the focal adhesions. Scale bar = 5 µm (**c**) Quantification of the number of adhered cells after 2 h seeding in plastic comparing the L-MSCs cells harvested from the hydrogels after 7 days of culture and the cells cultured in conventional 2D plastic substrate. Mean ± standard error (SE), n = 5 (**d**) Quantification of the focal adhesion length of the immunohistochemistry images of the actin and paxillin staining for the harvested and control cells after 2 h of reseeding them in plastic. Mean ± SE, n = 20 (**e**) qRT-PCR quantification of the expression of CXCR4 in the harvested L-MSC cells from the LC-L-ECM and HC-L-ECM structures after 7 days of 3D culture respect to the cells cultured in conventional 2D plastic substrate. Mean ± SE, n = 5.

Effective cell adhesion (Figure 3b) and length of focal adhesions (Figure 3c) were each visibly increased both in cells cultured within the LC-L-ECM and HC-L-ECM hydrogels as compared with the 2D control culture. Quantification of the images (Figure 3d,e) showed a statistically significant effect of the hydrogel culturing on the adhesion properties of L-MSCs. Indeed, as compared with the 2D control culture, between 6- and 10-fold more cells adhered to the TCP in the first 2 h (*p* value of 0.005 and *p*-value < 0.001 for cells harvested from LC-L-ECM and HC-L-ECM scaffolds, respectively). Moreover, focal adhesion lengths were more than two-fold greater than in cells cultured in 2D (*p*-value < 0.05 for cells harvested from both scaffolds). Quantification of the expression of CXCR4 in the harvested L-MSC cells from the LC-L-ECM and HC-L-ECM (Figure 3e) also showed a significant increase in the cells cultured in both high- (more than 20-fold increase, *p*-value of 0.004) and low-concentration hydrogels (6-fold increase, *p*-value of 0.007).

### 3.7. Immunomodulatory Assays and Cell Morphology

**Figure 4:**
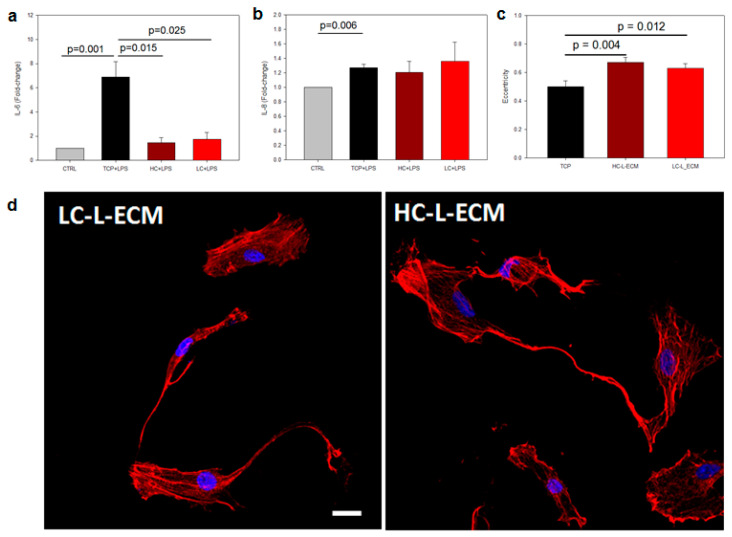
Immunomodulatory potential of MSCs. (**a**,**b**) Enzyme-linked immunosorbent assay (ELISA) quantification of the secretion of interleukin-6 (IL-6) and IL-8 cytokines in the co-cultures of L-MSCs and small airway epithelial cells (SAECs) after lipopolysaccharides (LPS) challenge. Mean ± SE, n = 5 (**c**) Quantification of cell eccentricity to characterize their morphology. Mean ± SE, n = 20 (**d**) Representative images for the actin (red) and nuclei (blue). Scale bar: 10 µm.

Cells harvested from the hydrogels showed a reduced secretion of IL-6 when cocultured with HSAECs after the LPS challenge, showing values similar to the control group and significantly decreased when compared to L-MSCs cultured in standard TCP (Figure 4a). No differences were observed in the secretion of IL-8 (Figure 4b). Both cells cultured in high- and low-concentration hydrogels presented a more elongated morphology, with significant differences found in the quantification of cell eccentricity (Figure 4c).

## 4. Discussion

The present study shows that 3D culturing L-MSCs within lung ECM hydrogel is a promising strategy to study fundamental properties of MSCs and to improve the therapeutic potential of these cells. Specifically, in vitro results show that L-MSCs cultured within lung ECM scaffolds present higher adhesive properties compared to cells cultured in standard TCP substrates in 2D. Moreover, cells cultured in 3D present an increase in CXCR4 expression and a reduction in the secretion of IL-6 cytokine when challenged in a co-culture in vitro model of lung injury. The development of a bioprinting strategy for the lung hydrogel-based scaffolds allows high-throughput fabrication of the 3D cultures.

The optimization of the hydrogel preparation method described herein, by solubilizing the powder at higher concentrations than previously published works, produced self-standing 3D scaffolds which are compatible with bioprinting technology, thus opening the possibility for high-throughput automated biofabrication of the 3D cultures, thus reducing the variability respect to manual procedures. Decellularized ECM-derived bioinks, due to their poor viscoelastic properties, commonly need to be mixed with other materials (alginate is the most used [50,51]) or methacrylated alginate [52,53] to be effectively bioprinted [54]. The main reason is that the concentration at which the ECM powder can be solubilized is usually low, as the most extended protocol is the developed by Pati and Cho [34] where the powder is solubilized at around 3 mg/mL and then the use of polycaprolactone polymer (PCL) is needed to support the structures. By raising the concentration of the ECM, and optimizing the digestion procedure, the developed structures present mechanical properties closer to the native ECM. Shortening the digestion time with respect to the standard protocols (48–72 h) has recently been shown to have a major impact on the hydrogel architecture and mechanical properties, and positively affects the viability of the cultured cells [55]: digestions shorter than 24 h result in highly branched and stiffer hydrogels, while the vast majority of the proteins involved in the gelation have been already solubilized. The particle size of the powder obtained after the cryogenic milling has a major impact in the digestion time needed to ensure the ECM is solubilized, and it should be optimized depending on the tissue source. For human lungs, it has been reported that after 72 h of digestion at 20 mg/mL there is still an insoluble fraction that should be removed by centrifugation [36], and this is a common procedure for ECM hydrogels’ preparation from other tissue sources. This fact makes difficult the estimation of final concentration of the ECM.

Analysis of SEM images and AFM mechanical data showed that the physical properties of the developed lung ECM scaffolds were quite similar to telocollagen hydrogels, which are known to be the stiffest ones among the different types of type I collagen structures [56]. No differences were observed in the SEM images between the different groups. Fiber diameter was not expected to change as it is formed by the collagen I fibrils which have been reported to be in the 100 nm dimeter range [57], hence the results obtained are consistent. Differences in fiber density related to different concentrations in the preparation of the hydrogel were expected. Nevertheless, the sample preparation method for SEM probably made the fibers to collapse towards the surface that was imaged, hiding this effect which is expected to appear in the non-treated 3D structures. Microscale mechanics measured with AFM, on the other hand, showed the expected difference depending on the concentration on the hydrogel. AFM is considered one of the best techniques to assess the mechanical properties of scaffolds at the length scale at which cells probe their microenvironment [58,59]. Results showed that the properties of HC-L-ECM were closest to those found in the lung parenchyma when thick strips were measured [60] and in the order of the reported values for thin slices [61]. Rheometry data showed that the viscosity of the L-ECM based bioinks is lower than the one observed in telocollagen, and more important, rheological properties were stable over time when maintained at 4 °C, in contrast with findings in collagen pregels, which made the setup for 3D bioprinting of the latter difficult. Indeed, changes in viscosity of the pregel during the bioprinting process results in the reduction of the fluid flow through the nozzle if the applied pressure is maintained constant. Therefore, for fluids with viscosity-changing over time, a complex control strategy for adapting the printhead pressure would be required, while ECM-based pregels can be bioprinted at constant pressure.

Cells could be easily cultured in the gels and showed no specific problems within the scaffolds after bioprinting, which confirms that the low pressures exerted by extruding the bioink in the pregel phase do not affect cells as they are similar to the pressures exerted in common cell culture protocols with a pipette [62]. Moreover, 3D confocal images showed a good distribution of the cells in the Z direction: cells within the bioink in the pregel phase do not massively precipitate towards the bottom of the structure during the printing or gelation time, which is a problem that could appear when using low viscosity bioinks [63]. As was observed in the contraction assays in the 3D cultures, L-MSCs interacted with the ECM hydrogel, which is the expected behavior for mesenchymal cells in collagen-based hydrogels [64]. The spread shape of cells observed in the immunohistological images of cells cultured inside the hydrogel suggests integrin-mediated cell attachment [65]. This cell–matrix interaction, which is a requisite for cells to respond to mechanical forces [66], is known to activate several pathways in the MSCs [67] such as the Hippo pathway. Indeed, it has been reported that the downregulation of this pathway is involved in improving therapeutic potential of MSCs for the treatment of acute lung injury [68,69]. The SDF1-CXCR4 pathway, highly related with the Hippo one [70], has been shown to have an impact in the immunomodulatory properties of MSCs [71] as well as increase the adhesion capacity of the cells [72,73]. Interestingly, quantification of the adhesion to the TCP of the harvested L-MSCs in comparison with the 2D control evidenced that cells cultured in the 3D lung ECM hydrogels had their adhesive properties increased. These results were confirmed by the quantification of the focal adhesions cells formed when plated on TCP. CXCR4 quantification by qPCR also confirmed the increased in effective adhesive properties of the cells which were cultured in 3D within the L-ECM hydrogels, suggesting that the SDF1-CXCR4 pathway is altered when cells are cultured within the hydrogels. Significant differences were found between the cells cultured in low- and high-concentration hydrogels. The fact that the mechanical properties of the HC-L-ECM hydrogels are closer to the native lung, suggests that the stiffest hydrogels could be best suited to study the behavior of cells for therapeutic applications.

The developed bioink allows cell passage in 3D as it is undertaken in standard 2D TCP cultures, as cells can be harvested from the scaffolds. The morphology of cells that were previously cultured within the scaffolds was markedly different from the cells cultured in TCP, which are commonly more spread. These differences in morphology (increased elongation) have been shown to predict an increased immunosuppressive capacity of MSCs [43], with the eccentricity being a critical parameter for the determination of cell shape. These differences in morphology were correlated with the experiments performed for an in vitro ARDS model in coculture of the MSCs with HSAECs subjected to an LPS hit. Results showing a marked decrease in IL-6 secretion and no significant differences in the secretion of IL-8 are in agreement with results reported for clinical trials with MSC therapy for ARDS [74]. These results reinforce the hypothesis that culturing MSCs in the developed scaffolds could unravel fundamental aspects about their immunomodulatory properties and thus in their therapeutic potential.

The bioprintability of the lung ECM hydrogels opens the door for high-throughput production of cell-laden scaffolds to achieve the number of 3D cultured cells needed to study them for therapeutic purposes [75], with respect to common bioreactors for culturing cells or lab-on-a-chip settings, which still are in the proof-of-concept phase due to scalability issues [76].

## 5. Conclusions

Results from the present study show that decellularized porcine lung hydrogels, with no external crosslinking, can be bioprinted and used as a 3D scaffold for studying mechanisms related to the therapeutic potential of L-MSCs. We have characterized hydrogel properties and we have conducted in vitro experiments that show good cell viability and potential modulation in their therapeutic potential. By using higher hydrogel concentrations through optimizing the cryogenic milling and solubilization processes, hydrogel structures are self-standing and the cells can be harvested several days after 3D culturing within the bioprinted scaffolds. Recent works have investigated the therapeutic properties of MSCs in respiratory diseases, but most of them have been carried out with cells from other sources (mainly bone marrow and adipose tissue). Understanding the interaction between lung MSC resident cells and their ECM is an important step towards answering fundamental questions of cell–matrix crosstalk, and potentially useful for the preconditioning of cells for therapeutic use. In vitro results on cell adhesion and the model of ARDS suggest that the cell immunomodulatory effect in vivo could be increased, especially when using the stiffer hydrogel scaffolds with mechanical properties more similar to the native lung parenchyma. Therefore, the data presented herein provide a solid background for further research in ECM-derived bioprintable hydrogels for the high-throughput production of scaffolds to study MSCs cells for therapeutic purposes.

## Data Availability

Data supporting the findings of this study are available from the corresponding author upon reasonable request.
